# ANCA-associated vasculitis in Ireland: a multi-centre national cohort study

**DOI:** 10.12688/hrbopenres.13651.1

**Published:** 2022-12-01

**Authors:** Jennifer Scott, Eithne Nic an Ríogh, Shamma Al Nokhatha, Cliona Cowhig, Alyssa Verrelli, Ted Fitzgerald, Arthur White, Cathal Walsh, Louis Aslett, Declan DeFreitas, Michael R. Clarkson, John Holian, Matthew D. Griffin, Niall Conlon, Yvonne O’Meara, Liam Casserly, Eamonn Molloy, Julie Power, Sarah M. Moran, Mark A. Little

**Affiliations:** 1 Trinity Health Kidney Centre, Trinity College Dublin, The University of Dublin, Dublin, D02 PN40, Ireland; 2 Department of Nephrology, Beaumont Hospital, Dublin, D09 V2N0, Ireland; 3 Department of Nephrology, Cork University Hospital, Cork, T12 DC4A, Ireland; 4 Department of Statistics, Trinity College Dublin, The University of Dublin, Dublin, D02 PN40, Ireland; 5 Department of Mathematics and Statistics, University of Limerick, Limerick, V94 T9PX, Ireland; 6 Department of Mathematical Sciences, Durham University, Durham, DH1 3LE, UK; 7 Department of Nephrology, St. Vincent’s University Hospital, Dublin, D04 T6F4, Ireland; 8 Department of Nephrology, University Hospital Galway, Galway, H91 YR71, Ireland; 9 Department of Immunology, St. James’s Hospital, Dublin, D08 NHY1, Ireland; 10 Department of Nephrology, Mater Misericordiae University Hospital, Dublin, D07 R2WY, Ireland; 11 Department of Nephrology, University Hospital Limerick, Limerick, V94 F858, Ireland; 12 Department of Rheumatology, St. Vincent’s University Hospital, Dublin, D04 T6F4, Ireland; 13 Vasculitis Ireland Awareness, Dublin, Ireland

**Keywords:** ANCA-associated vasculitis, registry, outcomes, death, end-stage-kidney-disease, urine soluble CD163 (usCD163)

## Abstract

**Background:** Antineutrophil cytoplasmic antibody (ANCA)-associated vasculitis (AAV) is a rare multisystem autoimmune disease. There is a need for interoperable national registries to enable reporting of real-world long-term outcomes and their predictors in AAV.

**Methods: **The Irish National Rare Kidney Disease (RKD) registry was founded in 2012. To date, 842 patients with various forms of vasculitis have been recruited across eight nephrology, rheumatology and immunology centres. We focus here on patient- and disease- characteristics, treatment and outcomes of the 397 prospectively recruited patients with AAV.

**Results:** Median age was 64 years (IQR 55–73), 57.9% were male, 58.9% had microscopic polyangiitis and 85.9% had renal impairment. Cumulative one- and five-year patient survival was 94% and 77% respectively. Median follow-up was 33.5 months (IQR 10.7–52.7). After controlling for age, baseline renal dysfunction (p = 0.04) and the burden of adverse events (p <0.001) were independent predictors of death overall. End-stage-kidney-disease (ESKD) occurred in 73 (18.4%) patients; one- and five-year renal survival was 85% and 79% respectively. Baseline severity of renal insufficiency (p = 0.02), urine soluble CD163 (usCD163) (p = 0.002) and “sclerotic” Berden histological class (p = 0.001) were key determinants of ESKD risk.

**Conclusions:** Long-term outcomes of Irish AAV patients are comparable to other reported series. Our results emphasise the need for personalisation of immunosuppression, to limit treatment toxicity, particularly in those with advanced age and renal insufficiency. Baseline usCD163 is a potential biomarker for ESKD prediction and should be validated in a large independent cohort.

## Introduction

Antineutrophil cytoplasmic antibody (ANCA)-associated vasculitis (AAV) is a rare multisystem autoimmune disease, characterised by necrotising pauci-immune small-vessel vasculitis. It comprises three clinico-pathological syndromes: granulomatosis with polyangiitis (GPA), microscopic polyangiitis (MPA) and eosinophilic granulomatosis with polyangiitis (EGPA). The estimated overall annual incidence of AAV is 13–20 cases/million ^1^ with a prevalence of 300–421 cases per million ^2^.

Significant treatment advances have occurred over the past 70 years with the advent of corticosteroid use in the 1950s, followed by the addition of cyclophosphamide in the 1960s, resulting in an improvement in two-year survival from 20 to 80% ^3^. The introduction of ANCA testing in the 1980s improved detection of AAV and augmented disease awareness. With the achievement of remission in the majority, accompanied by an improvement in immediate survival, focus has now shifted to examining long-term outcomes, influenced by both treatment and the disease itself. AAV still carries a 2.7-fold increased risk of death compared to the general population ^4^. Immunosuppression used to induce and maintain remission is a double-edged sword with >80% experiencing adverse events ^5^. Given the relapsing and remitting nature of AAV, short randomised controlled trials (RCTs) are unable to provide robust evidence on longer-term outcomes, such as long-term treatment safety and efficacy in the biologic era. Additionally, RCTs do not provide information on diverse populations who may not consent to inclusion in trials, such as those with very severe disease admitted to ICU at presentation, older patients, marginalised populations and pregnant women. This knowledge gap has prompted the development of longitudinal vasculitis registries, including at least eight in Europe ^6^. Increasingly, patient registries, linked to biobanks, are being used to facilitate longitudinal cohort studies in rare diseases, with a recent European effort to ensure standardisation and interoperability, via initiatives from FAIRVASC and the European Vasculitis Society (EUVAS) registries group ^6^. This will allow collaborative research using aggregated data to analyse critical outcomes at a larger scale with sufficient power.

The Irish Rare Kidney Disease (RKD) registry and biobank was created in collaboration with the Irish national vasculitis patient organisation ( https://vasculitis-ia.org/) in 2012 with the aim of collating clinical data and bio-samples from patients with vasculitis, and from disease and healthy controls on a national level. Importantly, this registry included, from inception, input from patient advocates (Vasculitis Ireland Awareness), recruitment during acute hospital admissions (allowing for pre-treatment data and sampling) as well as the involvement of a range of specialists including nephrology, rheumatology and immunology, allowing recruitment of broader clinical phenotypes. The current study arose from a need to appraise current practice and outcomes in the real-world setting. It aims to describe the long-term outcomes and potential prognostic factors at presentation in this large multicentre prospectively recruited cohort of AAV patients in Ireland.

## Methods

### Study participants

**(A) RKD registry.** The Irish Rare Kidney Disease (RKD) registry and biobank was established in 2012, recruiting from eight clinical centres (Extended data: Supplementary Table 1) ^7^. All patients >16 years of age with prevalent vasculitis were eligible for recruitment and diagnoses were assigned according to the 2012 Chapel Hill Consensus nomenclature ^8^. Central ethical approval was granted by the Tallaght University Hospital/St. James’s Hospital Joint Research Ethics Committee (reference 2019-08 List 29 (07)) on 30 ^th^ August 2019, and locally by each study site, and all participants provided written informed consent. Central storage of anonymised registry data is hosted on a secure password-protected web application (REDCap: https://www.project-redcap.org/) ^9, 10^.

**(B) Prospective AAV cohort.** In this paper we report in detail on participants with definite AAV defined by relevant clinical features of GPA, MPA or EGPA ^8^, with either positive anti-myeloperoxidase (MPO) or anti-proteinase 3 (PR3) serology ^11^ and/or diagnostic histopathology. Patients with secondary vasculitis or dual anti-glomerular basement membrane disease were excluded from this analysis ^12^. Patients who were positive for both MPO- and PR3-ANCA (n = 5) were classified according to whichever titre was higher ^13^. Analysis was restricted to only those diagnosed from 1 ^st^ January 2012 onwards, to eliminate retrospective recruitment bias (Extended data: Supplementary Figure 1) ^7^.

### Study assessments

The following data were collected prospectively: demographics, date of diagnosis and symptom onset, vasculitis characteristics (diagnostic subtype, organ involvement, ANCA specificity), treatment, histopathology, radiology, complications and follow-up clinical encounters (including disease activity assessed by the Birmingham Vasculitis Activity Score version 3 (BVAS v3) ^14^), urinalysis, pertinent laboratory data and exploratory biomarkers. Encounters were usually recorded at three-monthly clinic intervals and at the time of relapse, with a targeted annual minimum. Urine soluble CD163 (usCD163) was measured if clinically indicated in a central clinical laboratory using an accredited ELISA (Euroimmun, GMBH). The observation period was from the date of diagnosis to the occurrence of the event of interest (end-stage kidney disease (ESKD), death or last follow-up). Data collection is in agreement with the core dataset and interoperability principles outlined by EUVAS ^6^.

### Definitions

The degree of certainty in the AAV diagnosis was estimated using a novel pragmatic diagnosis confidence matrix (Extended data: Supplementary Figure 2) ^7^; only participants with a “definite” diagnosis were included in the prospective AAV cohort. ESKD was defined by a consensus EUVAS decision as the commencement of renal replacement therapy for at least 90 days (or death within 90 days), sustained estimated glomerular filtration rate <15ml/min and/or renal transplantation. The estimated glomerular filtration rate (eGFR, ml/min/1.73m ^2^) was calculated using the CKD-EPI formula. The ‘combined burden of events’ (CBOE) score is a representative summary variable to describe cumulative adverse events based on the Common Terminology Criteria for Adverse Events (CTCAE) and sub-categorised into “infection”, “leucopenia” and “other”, each rated on a score of 1–4 based on severity ^5^.

### Statistical analysis

Primary outcomes were time to ESKD and time to death. A composite of time to ESKD or death and factors associated with the occurrence of adverse events were also investigated. Continuous variables are reported as mean (standard deviation, SD) or median (interquartile range, IQR, if not normally distributed), and compared using the independent sample t-test or Mann-Whitney U test, respectively. Categorical variables are summarised by frequency and percentage (%) and compared using the chi-square test. Imputation was performed, using the *mice* R package (Version 3.13.0) ^15^, to estimate missing (9.1%) eGFR values ^16^. Survival probability was determined using Kaplan-Meier survival analysis. Analyses were censored for death or last follow-up and considered, firstly, the events only within the first year and, secondly, the events over the entire period of observation. Between group comparisons were performed using the log-rank test. Uni- and multivariate Cox regression analyses were performed separately to investigate predictors of mortality, ESKD, and the composite of ESKD/death. A backward stepwise method, in conjunction with a priori knowledge of significant confounders, was used to identify the final independent risk factors in the adjusted models. Potential confounders included both patient- and disease- characteristics: age and eGFR at diagnosis, sex, AAV phenotype, ANCA serology, organ involvement and CBOE score (or its components). Diagnosis BVAS was excluded from models due to its degree of missingness (28.5%). “Renal involvement” as a binary variable was also excluded due to strong correlation with diagnosis eGFR. The ‘ *surv_cutpoint’* function of the *survminer* R package (Version 0.4.9) ^17^ was used to identify the optimal binary cut-point for continuous predictors of importance, based on the maximally selected log-rank statistic ^18^. Cox proportional hazard ratios (HR) with 95% confidence intervals (95% CI) are reported, after evaluating the weighted and scaled Schoenfeld residuals to ensure the proportional hazards assumption was met. We performed multivariate logistic regression to identify key predictors of a CBOE score >8. Gender, AAV phenotype and induction treatment were included in this analysis as fixed factors, while diagnosis age and eGFR were continuous covariates. A two-tailed P value <0.05 was considered statistically significant. All statistical analyses were performed using R (Version 4.0.4). All survival analyses were performed using the *survival* R package (Version 3.2-11) ^19^.

## Results

### Participant characteristics

**(A) RKD registry.** As of 31 ^st^ May 2021, 842 unique patients with vasculitis were recruited (Extended data: Supplementary Table 2) ^7^. Most were Caucasian (97.7%) with a median diagnosis age of 59 years (IQR 48–69) and a male: female ratio of 1.2: 1. The commonest diagnosis was AAV (82.3%). A total of 644 (76.5%) underwent diagnostic biopsy, of which 532 (63.2% of all cases) showed definitive vasculitis. According to a diagnosis confidence matrix (Extended data: Supplementary Figure 2) ^7^, 750 (89.1%), 47 (5.6%) and 45 (5.3%) had a definite, possible and probable diagnosis confidence, respectively.

**(B) Prospective AAV cohort.** Of the 612 patients with a definite AAV diagnosis, 397 were prospectively recruited from 2012 onwards (Extended data: Supplementary Figure 1) ^7^; median age at diagnosis was 64 years (IQR 55–73) and 230 (57.9%) were male. The most observed phenotype was MPA (58.9%) and most (341, 85.9%) had renal involvement. Median baseline eGFR was 28 ml/min/1.73m ^2 ^(IQR 13–62). Median duration from symptom onset to diagnosis was 9.4 weeks (IQR 4.3–25.6) and follow-up continued for a median of 33.5 months (IQR 10.7–52.7). Complete patient- and disease-characteristics, including induction and maintenance treatment, are detailed in Table 1 and Extended data: Supplementary Table 3 ^7^.

**Table 1.** Baseline characteristics of the AAV cohort. Diagnosed with ‘definite’ AAV from 1st January 2012 onwards and recruited to the RKD registry before 31st May 2021. ‘Other’ Induction treatment includes AZA, MMF, MTX or GCC only. ‘Other’ Maintenance treatment includes: 1 <= of AZA, MMF, MTX, Tacrolimus, Ciclosporin, Cyclophosphamide, Mepolizumab, Ustekinumab, Hydroxychloroquine, Belimumab or unknown maintenance therapy. See Extended data: Supplementary Table 3 for further details on maintenance RTX group. Missing data: Induction treatment N = 6. AAV = ANCA associated vasculitis, eGFR = estimated Glomerular Filtration Rate (CKD-EPI), BVAS = Birmingham Vasculitis Activity Score, CYC = Cyclophosphamide, RTX = Rituximab, AZA = Azathioprine, MMF = Mycophenolate mofetil, GCC = Glucocorticoids. VINE = Vasculitis Ireland Network. IQR = interquartile range; SD = Standard deviation

Variable	Results
***n***	397
**Age at diagnosis (years, median (IQR))**	64 [55, 73]
**Male (N (%))**	230 (57.9)
Ethnicity* (N (%))	
White	392 (98.7)
Asian	5 (1.3)
***AAV Phenotype (N (%))***	
Granulomatosis with polyangiitis (GPA)	149 (37.5)
Microscopic polyangiitis (MPA)	234 (58.9)
Eosinophilic granulomatosis with polyangiitis (EGPA)	14 (3.5)
***ANCA Serotype (N (%))***	
Myeloperoxidase	214 (53.9)
Proteinase-3	172 (43.3)
ELISA negative	10 (2.5)
No ELISA performed	1 (0.3)
***Organ involvement (N (%))***	
Kidney	341 (85.9)
Lung	199 (50.1)
Musculoskeletal	137 (34.5)
Ear, Nose and Throat (ENT)	134 (33.8)
Mucocutaneous	71 (17.9)
Nervous	46 (11.6)
Gastrointestinal	17 (4.3)
Cardiovascular	11 (2.8)
**eGFR at diagnosis (ml/min/1.73 m ^2^, median (IQR))**	28.00 [13.00, 62.00]
***Induction treatment (N (%))***	
Cyclophosphamide	200 (51.2)
Rituximab	104 (26.6)
Cyclophosphamide & rituximab	28 (7.2)
Other	59 (15.1)
***Maintenance treatment (N (%))***	
Azathioprine	141 (35.5)
Rituximab +/- 1<= agent(s)	80 (20.2)
MMF	26 (6.5)
Glucocorticoids only	31 (7.8)
Other	78 (19.6)
Non	41 (10.3)
**End-stage kidney disease *(N (%))***	73 (18.4)
**Major relapse ( ^3^1) *(N (%))***	39 (9.8)
**Death at any time *(N (%))***	55 (13.9)
**Death within the 1 ^st^ year from diagnosis *(N (%))***	22 (5.5)
**Follow-up period (months, median (IQR))**	33.5 [10.7, 52.7]
**VINE Site *(N (%))***	258 (65.0)

### Patient survival in the first year from diagnosis (prospective AAV cohort)

**Figure 1.**
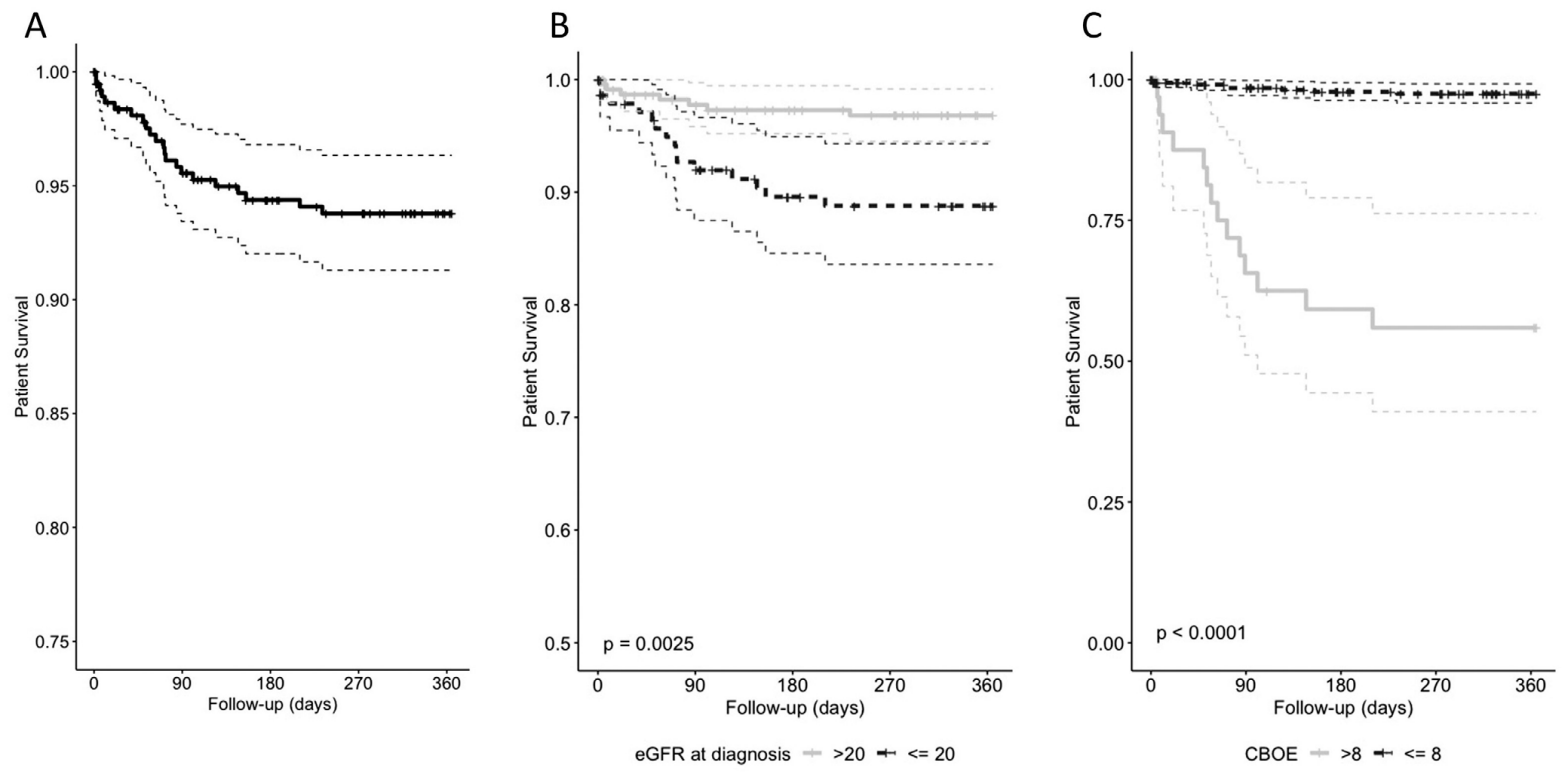
Factors associated with one-year mortality on univariate Kaplan-Meier analysis and compared using the log-rank test. Optimal cut-points were obtained for each factor using the survminer package in R studio. one-year mortality **a**). overall and by **b**). estimated Glomerular Filtration Rate (CKD-EPI, eGFR) <= / >20 ml/min/1.73 m2, and **c**). Combined Burden of Events score (CBOE) <=/>8.

In the first-year post diagnosis, twenty-two (5.5%) patients died, and actuarial survival was 93.8% (95% CI 91.3–96.3, Figure 1a). Infection was the leading cause of death (45.5%), followed by active vasculitis (18.2%, Extended data: Supplementary Table 4 ^7^). Age, degree of renal impairment at diagnosis, weighted infection and leucopenia adverse event scores, and cardiovascular involvement were independent predictors of one-year mortality on multivariate analysis ( Table 2). An eGFR cut-off of <20ml/min/1.73m ^2 ^identified those at highest risk of one-year mortality ( Figure 1b) ^18^. As the CBOE score rose, mortality increased. The optimal CBOE score (Extended data: Supplementary Figure 3) ^7^ mortality cut-off was >8 (Extended data: Supplementary Figure 4) ^7^, which was associated with a one-year survival probability of 55.9% (41.0–76.2) compared to 97.5% (95.8–99.2) for those with a score ≤8 ( Figure 1c). Renal dysfunction severity was an independent risk factor for a CBOE score >8 ( Table 3).

**Table 2.** Uni- and Multivariate Cox Proportional Hazards Models to investigate the factors associated with mortality within the first year from diagnosis. Multivariate analysis was performed using backwards elimination. There was no co-linearity (>0.7) observed between the predictive factors. AAV = ANCA associated vasculitis, GPA = granulomatosis with polyangiitis, PR3 = Proteinase-3 ANCA, eGFR = estimated Glomerular Filtration Rate (CKD-EPI), ml/min/1.73 m2, BVAS = Birmingham Vasculitis Activity Score, CYC = Cyclophosphamide, RTX = Rituximab, ENT = Ear, Nose and Throat, 95% CI = 95% Confidence Interval

	Univariate		Multivariate	
Explanatory variable	Hazard ratio (95% CI)	p Value	Hazard ratio (95% CI)	p Value
Age at diagnosis, years	1.09 (1.05–1.14)	<0.001	1.07 (1.02–1.12)	**0.009**
Female (Ref: Male)	1.79 (0.77–4.14)	0.174		
AAV Phenotype: Not GPA (Ref: GPA)	1.27 (0.52–3.10)	0.607		
ANCA: Not PR3 (Ref: PR3-ANCA)	1.67 (0.68–4.10)	0.262		
*Induction treatment (Ref: CYC)*				
RTX	1.42 (0.51–3.99)	0.506		
CYC & RTX	1.56 (0.34–7.20)	0.571		
Other	2.30 (0.77–6.86)	0.136		
eGFR at diagnosis	0.97 (0.95–0.99)	0.013	0.97 (0.94–0.99)	**0.026**
Weighted infection burden score	1.16 (1.11–1.21)	<0.001	1.11 (1.05–1.18)	**<0.001**
Weighted leucopenia burden score	1.37 (1.21–1.54)	<0.001	1.27 (1.11–1.44)	**<0.001**
Weighted adverse event (other) burden score	1.11 (1.07–1.15)	<0.001	1.05 (1.00–1.11)	0.07
*Organ Involvement*				
Lung	2.16 (0.88–5.31)	0.092		
Nervous	0.85 (0.20–3.65)	0.832		
ENT	0.71 (0.28–1.81)	0.474		
Cardiovascular	3.55 (0.83–15.18)	0.088	20.71 (3.84–111.81)	**<0.001**
Observations	397		395	
Concordance			0.91	

**Table 3.** Multivariate logistic regression to identify factors associated with a combined burden of adverse events (CBOE) score greater than 8, within the first year from diagnosis. There was no co-linearity (>0.7) observed between the predictive factors. N = 390 as one participant had missing age and six were missing induction treatment data. AAV = ANCA associated vasculitis, GPA = granulomatosis with polyangiitis, eGFR = estimated Glomerular Filtration Rate (CKD-EPI), ml/min/1.73 m2, 95% CI = 95% Confidence Interval

Explanatory variable	Odds ratio (95% CI)	p Value
Age at diagnosis, years	1.03 (1.00–1.06)	0.065
Female (Ref: Male)	1.10 (0.55–2.21)	0.78
AAV Phenotype: Not GPA (Ref: GPA)	0.95 (0.45–2.15)	0.90
eGFR at diagnosis	0.98 (0.96–0.99)	**0.01**

### Overall patient survival (prospective AAV cohort)

**Figure 2.**
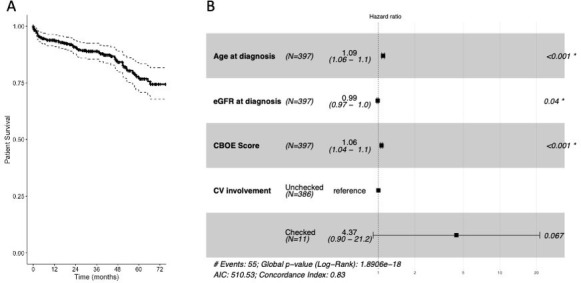
**a**). Kaplan-Meier curve demonstrating overall patient survival (solid line) with 95% CI (dashed lines), **b**). Forrest plot depicting the findings of a multivariate Cox Proportional Hazards Model to investigate the factors associated with overall mortality.

During the study period 55 patients (13.9%) died. Two- and five-year patient survival was 90.7% (87.7–93.9) and 76.8% (70.8–83.3), respectively ( Figure 2a). Figure 2b and Extended data: Supplementary Table 5 ^7^ summarise the factors associated with overall mortality. After controlling for age and other potential confounders, only eGFR at diagnosis and CBOE score were independently significantly associated with mortality. The CBOE mortality association was primarily driven by the cumulative infection score.

### Renal survival (prospective AAV cohort)

**Figure 3.**
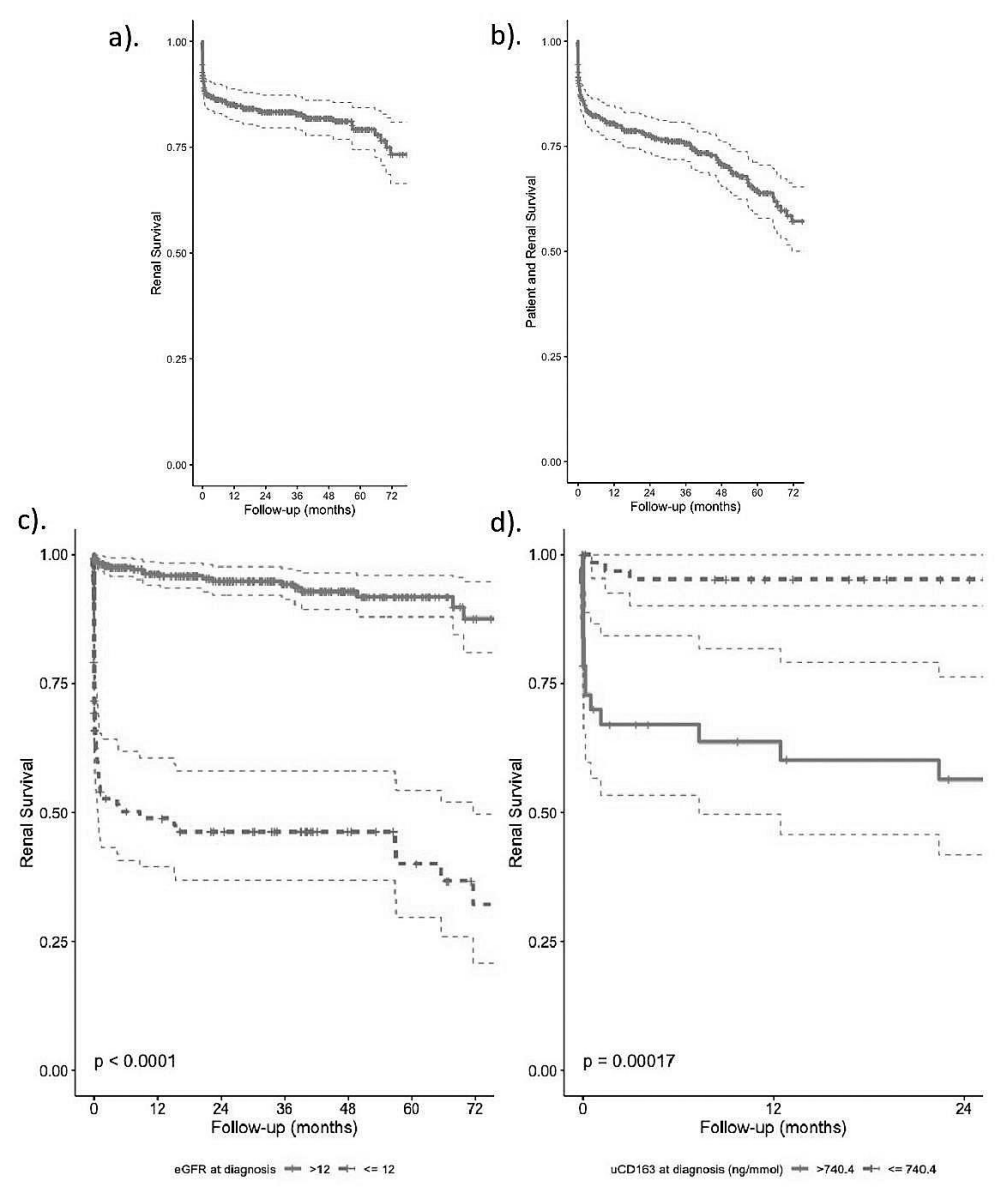
Factors associated with end-stage kidney disease (ESKD) on univariate Kaplan-Meier analysis and compared using the log-rank test. Optimal cut-points were obtained using the survminer package in R. **a**). Overall renal survival, **b**). ESKD-free patient survival, and renal survival stratified by **c**). estimated Glomerular Filtration Rate (CKD-EPI, eGFR) at diagnosis of <= / >12 ml/min/1.73 m2 and **d**). urine soluble CD163 at diagnosis (usCD163) of <= / >740.4 ng/mmol.

End-stage kidney disease (ESKD) developed in 73 patients (18.4%). 1-, 2- and 5-year actuarial renal survival was 85.1% (81.5–88.8), 83.3% (79.6–87.3) and 79.2% (74.4–84.4), respectively ( Figure 3a). ESKD-free patient survival at 1-, 2-, and 5-years was 80.6% (76.6–84.7), 77.7% (73.5–82.1) and 63.9% (57.9–70.5), respectively ( Figure 3b). At initial presentation 84 (21.1%) required renal replacement therapy, of whom 37 (44.4%) subsequently became dialysis independent. Of those who had renal involvement (86%), the median eGFR at diagnosis was 22 ml/min (IQR 11–42, Extended data: Supplementary Figure 5) ^7^. Factors independently associated with ESKD risk included degree of renal impairment and having >50% globally sclerosed glomeruli on kidney biopsy ( Table 4, model 2). usCD163 was measured at diagnosis in a subgroup of 103 patients, in whom usCD163 was tightly associated with ESKD, even after adjustment for eGFR ( Table 4, model 3). The optimal diagnosis eGFR and usCD163 cut-points which maximally split the cohort into low and high-risk groups of ESKD were 12ml/min/1.73m ^2^ and 740ng/mmol respectively ( Figure 3c–d and Extended data: Supplementary Figure 6a–b) ^7^. Patients with a ‘high’ (>740 ng/mmol) baseline usCD163 concentration were over five times more likely to develop ESKD, after adjusting for key confounders (Extended data: Supplementary Figure 7) ^7^.

**Table 4.** Uni- and Multivariate Cox Proportional Hazards Models to investigate the factors associated with end-stage kidney disease. ^a^Model 1 included baseline eGFR, adjusted for age at diagnosis, gender, AAV phenotype, ANCA serotype and the occurrence of a renal relapse (N = 397). ^b^Model 2 included baseline eGFR, adjusted for age at diagnosis, gender, AAV phenotype, ANCA serotype and Berden score on diagnostic renal biopsy (N = 244 as 153 participants did not have biopsy data). ^c^Model 3 included baseline uCD163, adjusted for baseline eGFR, age at diagnosis, gender, AAV phenotype and ANCA serotype (N = 103 as 294 participants did not have baseline uCD163 data). There was no co-linearity (>0.7) observed between the predictive factors included in the multivariate models. *Missing data: uCD163 N = 294, Berden score N = 153. AAV = ANCA associated vasculitis, GPA = granulomatosis with polyangiitis, PR3 = Proteinase-3 ANCA, eGFR = estimated Glomerular Filtration Rate (CKD-EPI), ml/min/1.73 m2, BVAS = Birmingham Vasculitis Activity Score, RRT = Renal Replacement Therapy, 95% CI = 95% Confidence Interval.

	Hazard ratio (95% CI, p Value)			
	Univariate		Multivariate	
Explanatory variable		Model 1 ^a^	Model 2 ^b^	Model 3 ^c^
Age at diagnosis, years	1.02 (1.00–1.04, p=0.054)	0.996 (0.98–1.02, p=0.708)	0.996 (0.97–1.02, p=0.709)	1.01 (0.98–1.05, p=0.498)
Female (Ref: Male)	0.68 (0.42–1.11, p=0.126)	0.66 (0.40–1.10, p=0.110)	0.65 (0.37–1.15, p=0.141)	0.17 (0.05–0.62, **p=0.007**)
AAV Phenotype: Not GPA (Ref: GPA)	2.78 (1.55–4.98, p=0.001)	1.34 (0.62–2.91, p=0.456)	0.94 (0.41–2.14, p=0.885)	1.28 (0.30–5.38, p=0.739)
ANCA: Not PR3 (Ref: PR3-ANCA)	1.87 (1.13–3.09, p=0.014)	0.86 (0.45–1.64, p=0.654)	0.69 (0.35–1.35, p=0.275)	2.09 (0.58–7.57, p=0.263)
eGFR at diagnosis	0.92 (0.89–0.94, p<0.001)	0.92 (0.89–0.94, **p<0.001**)	0.93 (0.90–0.96, **p<0.001**)	0.97 (0.94–0.995, **p=0.02**)
Required RRT at initial presentation	13.26 (8.01–21.96, p<0.001)	-	-	-
Renal relapse	0.74 (0.30–1.84, p=0.518)	0.57 (0.23–1.42, P=0.225)	-	-
Berden score on diagnostic renal biopsy (Ref: Focal)				
Crescentic	3.10 (1.16–8.32, p=0.024)	-	0.80 (0.27–2.36, p=0.683)	-
Mixed	3.62 (1.42–9.19, p=0.007)	-	2.13 (0.95–4.79, p=0.275)	-
Sclerotic	12.14 (4.93–29.86, p<0.001)		5.76 (2.61–12.71, **p=0.001**)	-
uCD163 at diagnosis (µg/mmol)	1.37 (1.13–1.68, p=0.002)	-	-	1.76 (1.24–2.50, **p=0.002**)
Observations	397 *	397	244	103
Concordance		0.87	0.84	0.80

## Discussion

We describe the Rare Kidney Disease (RKD) national Irish registry at the 10 ^th^ anniversary of its inception. This multicentre longitudinal cohort study aimed to characterise the Irish AAV cohort, describe their long-term outcomes and identify baseline predictors of these outcomes. The cohort comprises an ethnically homogeneous Caucasian population, with most displaying renal involvement and hence a slight MPA and MPO-ANCA predominance was observed. This contrasts to the GPA preponderance ^20– 23^ in most other European registries, which tend to have lower rates of renal involvement. Our cohort were typically older, in agreement with recent studies showing an older age at diagnosis for those with MPA ^21, 24, 25^. We noted a slight male preponderance, consistent with a prior meta-analysis of EUVAS RCTs ^22^, which differs from the equal gender split most commonly reported ^2, 20, 21, 23^). Notably, the diagnostic delay (median 9.4 weeks, IQR 4.3–25.6, N = 238) in our cohort was somewhat shorter than other reported series (median range 12–52 weeks ^21, 26– 29^).

Patient survival in our real-world cohort was comparable to the international literature, predominantly based on RCTs, which ranges from 80–97%, 80–85% and 70–80% for one, two and five years respectively ^2, 5, 22, 26, 30, 31^). One may have expected lower rates given our relatively older cohort with frequent renal insufficiency. We too noted a steep decline in survival in the initial 90 days, when maximal concomitant disease activity and immunosuppression occurs. Infection was the primary cause of death, consistent with previous reports ^5, 22, 26, 32, 33^). Multiple studies have demonstrated an inverse relationship between survival and the number of accrued infections, particularly in the first year ^31, 34^). In our study, the weighted infection and leucopenia scores, reflecting the accumulation of these adverse events, were the strongest predictors of one-year mortality. This highlights the growing need for personalisation of immunosuppression to simultaneously achieve disease control while avoiding unnecessary excess immunosuppression, in addition to steroid-sparing, borne out by the PEXIVAS trial ^35^. It also serves as a key reminder to clinicians to be alert to early infections and to routinely assess the need for infection prophylaxis against *Pneumocystis jirovecii* (PCP), fungi and viruses, as well as timely influenza, pneumococcal, hepatitis B virus and SARS-CoV-2 vaccination, as advised in guidelines ^36^. Notably, only one patient died from PCP in our cohort, which may signify the success of trimethoprim/sulfamethoxazole prophylaxis, which is cost-effective in AAV ^37^.

Our study supports the previously observed association between increasing age, impaired kidney function and early mortality ^5, 20, 21, 30, 33, 38– 41^). While most studies have previously attributed this to excess severe infection ^21, 31, 32, 42, 43^, we observed that advanced age and renal dysfunction are independent predictors, *after* adjustment for adverse events. Renal impairment results in immune dysfunction ^44^ and altered pharmacokinetics, limiting drug clearance and hence increasing the risk of infection and treatment toxicity ^45^. Our findings further support the use of dosing nomograms for cyclophosphamide based on age and eGFR to reduce treatment-induced leucopenia, thereby reducing mortality ^32, 45^. Future prospective research should explore whether a similar approach is necessary for rituximab, rather than the current fixed or weight-based dosing regimens.

Our study validates the use of the combined burden of events (CBOE) score ^5^, to predict early mortality in an independent real-world cohort. Building on the initial description, we identified 8 as an optimal cut-off score to stratify patients into low/high risk of early death. Like the original description, a score ≤8 was associated with <5% risk of death within the first year. This risk rose 16-fold with a score >8. Analogous to scoring BVAS and Vasculitis Damage Index (VDI) during routine follow-up, clinicians should consider monitoring the cumulative CBOE score. A rising score warrants active assessment and implementation of strategies to reduce further toxicity. We also confirmed that renal dysfunction severity is the strongest independent risk factor of the CBOE score, underpinning the critical importance of accounting for kidney function when prescribing immunosuppression ^5^. We found no statistical difference in the risk of a high CBOE score between the cyclophosphamide and rituximab groups, in keeping with the findings of the RAVE trial ^46^. Our findings emphasise that no induction regime is ‘safe’ and future research should focus on developing remission induction strategies that minimise toxicity, while maintaining similar efficacy.

Reported renal survival in AAV is variable, largely related to differing baseline patient and disease characteristics across cohorts. Our one-, two- and five-year renal survival estimates of 85.1%, 83.3% and 79.2% respectively are in line with other series ^26, 31, 47– 50^): 20–40% of AAV patients reach ESKD by 5–7 years. Aligned with our relatively short diagnostic delay, the baseline renal function (median eGFR 22 ml/min 1.73m ^2^) in those with renal involvement was comparable to international benchmarks (median eGFR range 12–25 ml/min/1.73m ^2^) ^51, 52^. Our results confirm the importance of baseline renal dysfunction ^26, 31, 32, 47, 49, 53^ and of histological evidence of renal scarring ^54^ in determining ESKD risk. Contrary to prior series, we did not find an association between ANCA serotype ^50^ or prior relapse ^47^ and ESKD, in multivariate analysis. Our group has recently demonstrated the benefit of usCD163, a glomerular macrophage marker, as a non-invasive tool for the diagnosis of AAV renal relapse ^55^. Another group highlighted its use in the prediction of doubling of serum creatinine in lupus nephritis ^56^. We demonstrate, for the first time, the potential use of baseline usCD163 in predicting ESKD at diagnosis, even after adjustment for renal insufficiency. We identified a cut-off of 740ng/mmol to stratify patients into low/high risk groups. The risk of ESKD was five-times higher for participants with a baseline usCD163 of >740.4ng/mmol compared with those below this cut-point. Once validated in independent cohorts, these findings will contribute to the personalisation of immunosuppression to minimise ESKD risk – a costly outcome to patients and society.

The main strengths of our study include the well-characterised national AAV cohort, recruited using uniform classification criteria. Our cohort does not exclude patients with severe disease, EGPA nor older people – commonly excluded in RCTs – and thus serves as a source of real-world data, which is important as outcomes differ to those reported from RCTs ^57^. The use of REDCap as the registry infrastructure, with standardised interoperable ‘instruments’ to enable uniform data collection is another key strength. Our registry formed the basis of the model EUVAS registry which is being applied in nascent vasculitis registries across Europe. This capability is in line with both the EUVAS ^6^ and European Reference Network initiatives ( http://rita.ern-net.eu/about-rita/mission-goals-and-objectives/) to align and integrate European registries, with the aim of improving both care and outcomes in rare disease.

We also acknowledge the limitations of our study. As with any observational research, missing data is always a challenge. For this reason, we were unable to include BVAS in multivariate models. Although all relevant specialties were represented, most participants were recruited through nephrology centres, reflected in the high degree of renal involvement, which limits the generalisability of our findings to non-renal populations. The strengths and limitations of CBOE score were previously discussed in detail ^5^. Additionally, the CBOE score was not adjusted for prophylaxis use. Induction treatment was included as a categorical variable – the variation in intensity of same (denoted by cumulative dosing) was not available. Going forward, we aim to capture the intensity of induction immunosuppression in our registry.

## Conclusions

In conclusion, we demonstrated that long-term outcomes from a real-world Irish cohort are similar to international standards. Our results highlight that despite improvement in outcomes over the last number of decades, treatment toxicity is an ongoing concern and individualisation of immunosuppression relative to disease severity and relapse risk remains a key unmet need. Our findings serve to remind clinicians that the accumulation of adverse events, both in the first year and beyond, particularly in older patients and those with renal insufficiency, is strongly associated with premature death and should be accompanied by increased monitoring and adjustment of immunosuppression to further limit treatment-related morbidity. The degree of renal impairment at presentation is also a key determinant in renal survival, hence early identification remains a critical goal. Baseline usCD163 shows promise as a biomarker for ESKD prediction. Future research should further explore this in the context of a multi-modal approach to personalisation of AAV treatment.

## Consent

Written informed consent for publication of the patients’ details was obtained from the patients.

## Data Availability

While the underlying patient data is pseudonymised, due to the rarity of ANCA-associated vasculitis, coupled with the identifiable nature of the data included in our analyses, it is not possible in practice to fully anonymise the dataset. Individuals could potentially be re-identified quite easily. Therefore, raw data must remain confidential and cannot be freely shared on an open platform. We would invite any potential research collaborations or data requests through the corresponding author, Professor Mark Little ( mlittle@tcd.ie), on reasonable request, as agreed by participants in their written informed consent (detailed on page 3: https://www.tcd.ie/medicine/thkc/assets/pdf/RKD-Vasculitis-Patient-PIL-ICF-Version-5-07AUG19.pdf). Requests will be considered on a case-by-case basis. This approach is endorsed in a recent publication in The Lancet, from another large Irish longitudinal cohort study, TILDA ^58^. Zenodo: Extended data for ‘ANCA-associated vasculitis in Ireland: a multi-centre national cohort study’. https://doi.org/10.5281/zenodo.7342934 ^7^ This project contains the following extended data: Supplementary Table 1: Rare Kidney Disease Registry and Biobank recruitment sites. Supplementary Table 2: Baseline characteristics of all vasculitis patients (recruited prior to 31 ^st^ May 2021). Supplementary Table 3: Breakdown of additional agents used for those patients receiving maintenance rituximab (n=80). Supplementary Table 4: Causes of death within the first year of observation in the AAV cohort diagnosed since 2012. Supplementary Table 5: Uni- and Multivariate Cox Proportional Hazards Models to investigate the factors associated with overall mortality. Supplementary Figure 1: Inclusion and exclusion criteria for the AAV patients included in the analysis of long-term outcomes. Supplementary Figure 2: Diagnosis confidence matrix. Supplementary Figure 3: Frequency and distribution of Combined Burden of Events (CBOE) score within the first year from diagnosis. Supplementary figure 4: Impact of the Combined Burden Of adverse Events (CBOE) on mortality. Supplementary Figure 5: Distribution of eGFR at diagnosis (ml/min/1.73/m2) in those with renal involvement: overall and stratified by VINE status. Supplementary Figure 6: Optimal cut-points of a). 12 ml/min/1.73m2 for eGFR at diagnosis and b). 740.4 ng/mmol for usCD163 at diagnosis were determined, using the *survminer* package in R to categorize patients into low and high risk of ESKD. Supplementary Figure 7: Forrest plot depicting the findings of a multivariate Cox Proportional Hazards Model to investigate the factors associated with end-stage kidney disease. usCD163 is included as a binary factor according to the optimal cut-point, leading to slightly different hazard ratios compared to Table 4. Data are available under the terms of the Creative Commons Attribution 4.0 International license (CC-BY 4.0)
