# Supplementary material for: ANCA-associated vasculitis in Ireland: a multi-centre national cohort study
Source: HRB Open Res. 2022 Dec 1;5:80. [Version 1] doi: 10.12688/hrbopenres.13651.1 (PMC10213823; doi:10.12688/hrbopenres.13651.1)
Supplement: Peer review history

# Peer review: Reviewer response for version 1

**DOI:** 10.21956/hrbopenres.14929.r33751

**Published:** 2023-05-25

**Authors:** Jessica Ryan

**Affiliations:** 1 Department of Nephrology, Monash Medical Centre, Monash Health, Melbourne, Vic, Australia

The authors should be congratulated on this publication. It reports on a large registry of AAV patients and is overall excellent.

 A few minor comments:

- With regards to the baseline characteristics of the AAV cohort, the percentage of AAV serologically negative patients seems low (only 2.5%) compared to other published series. Is there a reason for this? In addition the median duration from symptom onset to diagnosis is lower than expected. Can the authors postulate an explanation in the discussion?
- With regards to table 1, does major relapse refer to patients experiencing a relapse over what time frame? Are relapses able to be described in more detail?
- The way Rituximab maintenance is depicted in the table is confusing and took me a while to work out (after looking at the supplemental data).  
- Why was there no association between ANCA serotype or prior relapse and ESKD?

Is the work clearly and accurately presented and does it cite the current literature?

Yes

If applicable, is the statistical analysis and its interpretation appropriate?

Yes

Are all the source data underlying the results available to ensure full reproducibility?

Yes

Is the study design appropriate and is the work technically sound?

Yes

Are the conclusions drawn adequately supported by the results?

Yes

Are sufficient details of methods and analysis provided to allow replication by others?

Yes

Reviewer Expertise:

Glomerulonephritis, ANCA associated vasculitis

I confirm that I have read this submission and believe that I have an appropriate level of expertise to confirm that it is of an acceptable scientific standard.

# Peer review: Reviewer response for version 1

**DOI:** 10.21956/hrbopenres.14929.r33415

**Published:** 2023-05-25

**Authors:** Y.K. Onno Teng

**Affiliations:** 1 Department of Nephrology, Leiden University Medical Centre, Leiden, The Netherlands

This is a very nice overview of AAV patients in Ireland and adds an important piece of information to our perceptions of disease prevalence, incidence of a rare disease like AAV. It is also very informative to see confirmation of risk factors of mortality in this cohort with infections as the leading cause. These data prompt the field to think about improving treatment for this patients based on data-driven decisions, like this study. There is obvious unmet need in the intensive immunosuppression regimen used for remission induction of AAV.

 I think the manuscript would gain in impact if some of the supplementary analysis were moved to the main manuscript. There is very valulable information in Supplementary Table 5 and Supplementary Figure 7. Both provide additional information to the clinical practice as to which subgroup of patients is at risk for dying and which subgroup is at risk (or not) for EsKD.

Is the work clearly and accurately presented and does it cite the current literature?

Yes

If applicable, is the statistical analysis and its interpretation appropriate?

Yes

Are all the source data underlying the results available to ensure full reproducibility?

Yes

Is the study design appropriate and is the work technically sound?

Yes

Are the conclusions drawn adequately supported by the results?

Yes

Are sufficient details of methods and analysis provided to allow replication by others?

Yes

Reviewer Expertise:

Renal autommune diseases, glomerulonephritis, ANCA-associated vasculitis, lupus nephritis, membranoproliferative glomerulonephritis

I confirm that I have read this submission and believe that I have an appropriate level of expertise to confirm that it is of an acceptable scientific standard.
